# Projected shifts in the potential distribution of *Astilbe* under the SSP1-2.6 climate scenario

**DOI:** 10.3389/fpls.2026.1887603

**Published:** 2026-07-16

**Authors:** Jiayi Liu, Lei Zhang, Dongsheng Wang, Yanan Jing, Guojun Zhang, Beibei Cheng, Fusheng Wu

**Affiliations:** 1 College of Horticultural Science and Technology, Hebei Normal University of Science & Technology, Qinhuangdao, Hebei, China; 2 College of Horticultural Science and Technology, Hebei Key Laboratory of Horticultural Germplasm Excavation and Innovative Utilization, Qinhuangdao, Hebei, China; 3 Hebei Higher Institute Application Technology Research and Development Center of Horticultural Plant Biological Breeding, Hebei Normal University of Science & Technology, Qinhuangdao, Hebei, China; 4 Resource Conservation Institute, Shandong Provincial Forest and Grass Germplasm Resources Center, Jinan, Shangdong, China

**Keywords:** *Astilbe*, climate change, maximum entropy model, SSP1-2.6, suitable habitats

## Abstract

**Introduction:**

This study aimed to project the potential distribution and habitat suitability dynamics of seven *Astilbe* species in China from 2000 to 2080.

**Methods:**

MaxEnt modelling was employed under the ACCESS-CM2 climate model and the SSP1-2.6 emission scenario, integrating field survey data, herbarium records, and 23 environmental variables, under the assumption of static soil and elevation conditions.

**Results:**

The results indicated that annual precipitation (bio12) and elevation (DEM) had the strongest statistical associations with the geographic distribution of the genus, while temperature and soil factors played secondary roles. Under current climatic conditions, the core suitable areas are concentrated mainly in Northeast and Southwest China, with pronounced interspecific spatial differentiation: *A. rubra* had the greatest suitable habitat area, whereas *A. longicarpa* had the smallest. Under future scenarios, the total suitable habitat area of the six species expanded, with *A. longicarpa* being the sole exception. The direction of centroid displacement varied among species: *A. grandis*, *A. rivularis*, *A. longicarpa*, and *A. macroflora* shifted towards higher latitudes, whereas *A. chinensis*, *A. rubra*, and *A. macrocarpa* shifted towards lower latitudes. At the genus level, habitat redistribution exhibited pronounced interspecific variation and directional heterogeneity. *A. longicarpa* and *A. macroflora* were identified as the most potentially vulnerable species, given their extremely restricted suitable areas, the absence of highly suitable habitat under current conditions, and their continued confinement largely to Taiwan Province.

**Discussion:**

For these range-restricted taxa, strengthened population monitoring within existing native ranges and assessments of *ex situ* conservation feasibility are warranted. These findings provide spatially explicit information to guide conservation planning for medicinal plant resources under climate change.

## 1 Introduction

*Astilbe* Buch.-Ham. is a perennial herbaceous genus in the family Saxifragaceae. Plants in this genus contain bioactive compounds such as astilbin and bergenin and have been reported to possess a range of pharmacological properties, including antitumor effects, improved blood circulation, and expectorant activity (Yin et al., 2018). The genus comprises approximately 18 species worldwide, distributed mainly in Asia—particularly East Asia—and North America. Seven species occur in China, three of which are endemic. These species are found across both northern and southern China, with particularly high diversity in the eastern, central, and southwestern regions (Flora of China Editorial Committee and Chinese Academy of Sciences, 1992; Xu and Zang, 2015). The seven taxa in China are *A. chinensis*, *A. grandis*, *A. longicarpa*, *A. macroflora*, *A. rubra*, *A. macrocarpa*, and *A. rivularis* var. *rivularis* (Zheng, 2024). A phylogenetic study by Wen, based on plastid and ITS gene trees of *Astilbe*, revealed that *A. chinensis*, *A. grandis*, *A. longicarpa*, *A. macroflora*, *A. rubra*, and *A. macrocarpa* form Clade I, whereas *A. rivularis* constitutes Clade III, with the two clades being sister groups (Zhu et al., 2013).

Medicinal plant diversity (MPD) is a vital component of global plant diversity, yet climate change and overexploitation increasingly threaten the habitats of medicinal species. By altering temperature and precipitation regimes, climate change can directly redistribute species range, thereby affecting the availability and quality of medicinal plant resources. Although changes in growing conditions may also influence the accumulation of defensive secondary metabolites such as flavonoids and saponins, potentially reducing biomass and compromising yield stability (Mykhailenko et al., 2025), global warming further intensifies environmental stresses—including soil drought, salinization (Vaishnav et al., 2019), extreme precipitation (Trenberth, 2011), and pest and disease outbreaks (Kocmánková et al., 2009)—, which can drive range contractions and local extinctions and can pose severe risks to ecosystem stability and agricultural sustainability. In East Asia, these pressures are already evident: *Panax ginseng* is projected to undergo overall range reductions under future emission scenarios (Zhao et al., 2016), and climate aridification and warming are expected to decrease the suitable habitat of *Glycyrrhiza uralensis* in northwestern China and Mongolia, with some studies highlighting a growing risk of habitat fragmentation for this species (Zhang et al., 2026). However, comparable assessments remain scarce for many other herbaceous medicinal perennials in the region. Systematically projecting future shifts in habitat suitability is therefore critical for conservation and sustainable use planning.

Species distribution models (SDMs) combined with climatic data are widely used to predict potential species distributions (He et al., 2023). SDMs have become an important tools in ecology, biogeography, and evolutionary biology (Hirzel et al., 2002; Peterson et al., 2007; Zhang et al., 2008; Phillips et al., 2009; Morales et al., 2017). Common models include CLIMEX (Byeon et al., 2018), maximum entropy (MaxEnt) (Baldwin, 2009), GARP (Banks et al., 2009), ENFA (Morales et al., 2017), and the Bioclimatic Envelope Model (Pearson and Dawson, 2003). Among these, MaxEnt is among the most frequently applied SDMs because of its user−friendly interface, ability to perform well with small sample sizes, and generally high predictive accuracy (Hernandez et al., 2006; Merow et al., 2013). For example, Song et al. (2025) used MaxEnt to predict the current and future suitable habitats of *Ligustrum lucidum*; Zhang et al. (2025) employed an optimized MaxEnt model to assess the global potential habitat suitability of *Fusarium circinatum* and its response to future climate change; and De Barros et al. (2025) used MaxEnt to model the distribution of *Digitaria insularis* under various climate scenarios. Therefore, the MaxEnt model was selected in this study to predict the suitable habitats of *Astilbe* species. Additionally, Feng and Yue (2015) demonstrated that moderate drought causes irreversible damage to *A. grandis*, and through MaxEnt and GIS analyses, Que and Dai (2022) reported that precipitation, temperature, and elevation (DEM) are key factors affecting the distribution of *A. chinensis*. Based on these studies, we hypothesized that precipitation, temperature, and the DEM are the primary environmental factors that shape the geographic distribution of *Astilbe*. Species in this genus possess ornamental, medicinal, and ecological value.

The results of this study extend predictions of habitat suitability for the genus *Astilbe* from a single species (*A. chinensis*) to seven species, addressing a significant gap in the understanding of the habitat dynamics of the genus under future climate conditions. In contrast to the single−species, single−period analysis of Que et al (Que and Dai, 2022), this work integrates current climate data (2000–2024) with future projections for three time periods (2021–2040, 2041–2060, and 2061–2080) under the SSP1−2.6 scenario, employing a single climate model (ACCESS−CM2) and assuming static soil and elevation conditions. This multi−species, multi−temporal framework shifts the analytical perspective from a static retrospective view to a dynamic forwards−looking projection. Building on the foundational work of Que et al (Que and Dai, 2022), we selected seven species*—A. chinensis*, *A. grandis*, *A. rivularis*, *A. rubra*, *A. macrocarpa*, *A. longicarpa*, and *A. macroflora—*to (1) simulate their current and future suitable habitat ranges, (2) identify key environmental predictors, and (3) quantify spatial shifts in habitat suitability across time periods under a consistent scenario. Based on existing research and observed occurrence data, we inferred that temperature and precipitation are the dominant environmental factors associated with the distribution of *Astilbe* under a changing climate. Throughout the 2000–2080 period, a general trend of increasing temperature and precipitation is projected. Species whose distributions are statistically linked primarily to these factors are more likely to exhibit a projected northwards shift in their suitable habitat centroid. In this study, a genus−level analytical framework, employing MaxEnt models, was established to project climate−driven shifts in habitat suitability for *Astilbe* species. By resolving the full spectrum of responses—from narrowly endemic taxa to widely distributed congeners—the framework yields a generalizable conservation baseline for the East Asian flora and provides an evidence−based foundation for developing targeted conservation strategies.

## 2 Materials and methods

### 2.1 Data sources and processing

A total of 7,546 occurrence records were compiled for Astilbe species from four sources: field surveys conducted under the national resource inventory (**Supplementary Table 1**), the Chinese virtual herbarium (CVH; https://www.cvh.ac.cn/), the global biodiversity information facility (GBIF; https://www.gbif.org/), and published literature (Yuan et al., 2019) (**Supplementary Table 2**).

**Figure 1:**
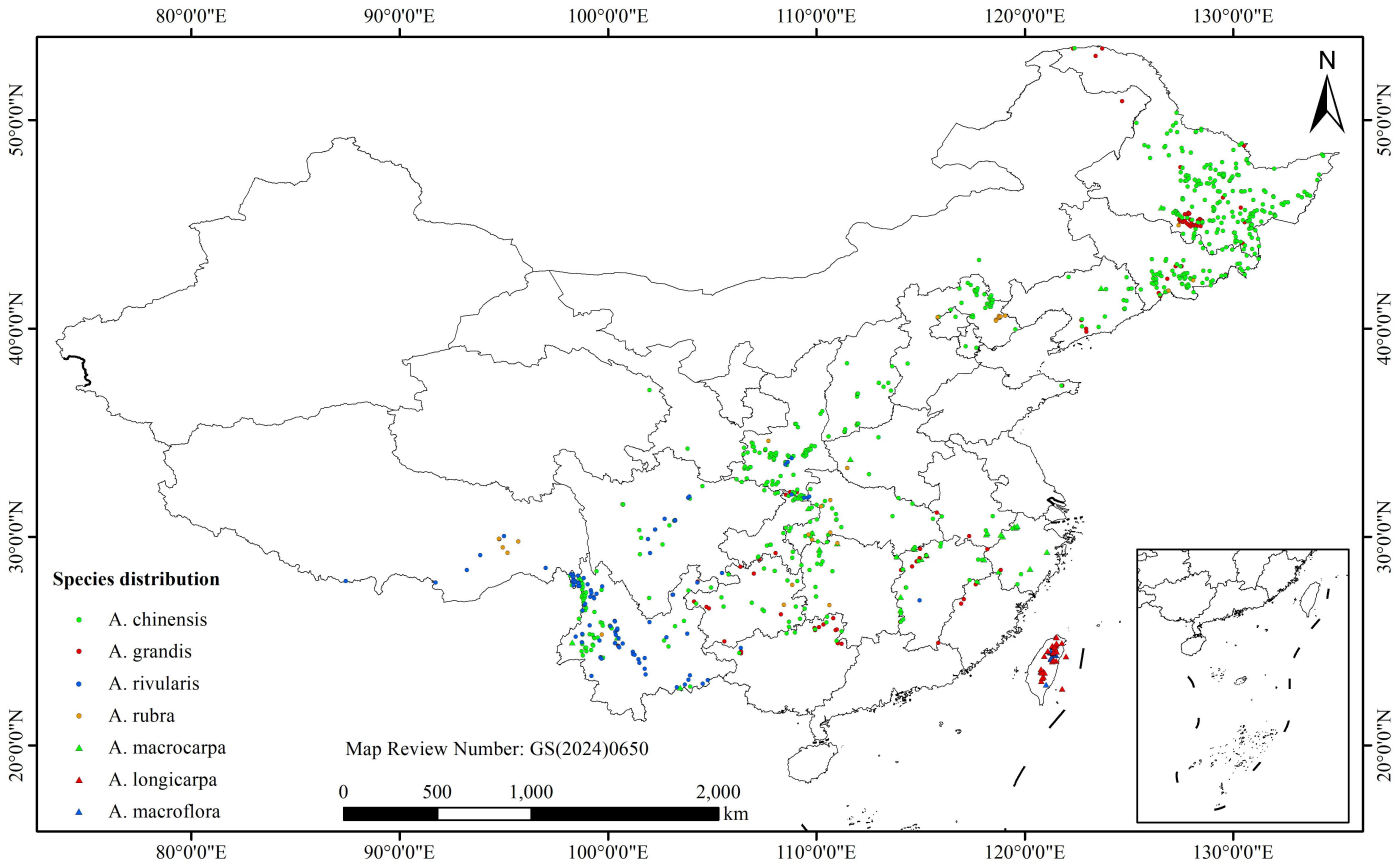
Distribution map of *Astilbe* in China.

To reduce the potential for sampling bias introduced by the integration of multisource data, all the occurrence records were first merged. Records with erroneous coordinates, duplicate entries, missing essential information, or those located outside the study area (i.e., outside China) were then removed, yielding a preliminarily screened dataset. To further minimize spatial clustering and potential sampling bias, spatial thinning was applied by enforcing a minimum distance of 3 km between retained occurrence points (Wang et al., 2017). Only records from the year 2000 onwards were kept, resulting in a final set of 932 occurrence records used for habitat suitability modeling (**Figure 1**).

This study included three categories of environmental predictors—climatic, edaphic, and topographic—for a total of 23 variables (**Table 1**). The DEM was obtained from the WorldClim database (https://www.worldclim.org/) (Fick and Hijmans, 2017). For soil data, soil variables were sourced from the Harmonized World Soil Database (HWSD; https://www.fao.org/soils-portal/en/).

**Table 1:** Environmental predictors: bioclimatic, soil, and topographic variables.

Factor code	Description	Unit or description
bio 1	Annual mean temperature	°C
bio 2	Mean diural rangemean of monthly	°C
bio 3	Isothermality (bio 2/bio 7)(×100)	°C
bio 4	Temperature seasonality (standard deviation x100)	°C
bio 5	Max temperature of warmest month	°C
bio 6	Min temperature of coldest month	°C
bio 7	Temperature annual range (bio 5-bio 6)	°C
bio 8	Mean temperature of wettest quarter	°C
bio 9	Mean temperature of driest quarter	°C
bio 10	Mean temperature of warmest quarter	°C
bio 11	Mean temperature of coldest quarter	°C
bio 12	Annual precipitation	mm
bio 13	Precipitation of wettest month	mm
bio 14	Precipitation of driest month	mm
bio 15	Precipitation seasonality (coefficient of variation)	mm
bio 16	Precipitation of wettest quarter	mm
bio 17	Precipitation of driest quarter	mm
bio 18	Precipitation of warmest quarter	mm
bio 19	Precipitation of coldest quarter	mm
clay	Clay content	%wt
silt	Soil silt content	%wt
sand	Soil sand and gravel content	%wt
Dem	Elevation	m

For current climate data, 19 bioclimatic variables representing current conditions were derived from the CRU TS v4.09 (climatic research unit time series) gridded dataset. We extracted four monthly variables—monthly mean minimum temperature (tmn), monthly mean maximum temperature (tmx), monthly mean temperature (tmp), and monthly total precipitation (pre)—for the years 2000, 2005, 2010, 2015, 2020, and 2024. For each year, the 19 bioclimatic variables (bio1–bio19) were computed independently following the standard WorldClim algorithms, and the multiyear means were then calculated to produce the current baseline (2000–2024) bioclimatic layers, hereafter referred to as the 2010s in the modelling workflow.

For, future climate data, future bioclimatic projections were obtained from the WorldClim CMIP6 database (version 2.1). Because *Astilbe* species are distributed primarily in East Asia (China, Japan, and the Korean Peninsula), and the ACCESS-CM2 model has been shown to perform well in simulating key climatic variables in this region (Ju et al., 2025), we selected it for our study, which focused on China. The model outputs were taken under the SSP1-2.6 scenario, which combines Shared Socioeconomic Pathway 1 (SSP1) with representative concentration pathway 2.6 (RCP2.6) and represents a sustainable development trajectory with low greenhouse gas emissions. Future climate conditions are represented by three 20-year climatological means: 2021–2040 (hereafter the 2030s), 2041–2060 (the 2050s), and 2061–2080 (the 2070s).

All the raster layers were resampled to a common spatial resolution of 30 arc seconds (~1 km at the equator) and aligned to the WGS 1984 geographic coordinate system. Data processing was performed in ArcMap 10.8 (Esri, Redlands, CA, USA). The national boundary vector of China was obtained from the Standard Map Service System of the Ministry of Natural Resources (National Public Service Platform for Geographic Information).

### 2.2 MaxEnt model construction and suitable area zoning

Habitat suitability models for the seven *Astilbe* species were constructed using the maximum entropy algorithm implemented in MaxEnt (version 3.4.3) by integrating species occurrence records with 23 environmental predictors. The model was run on the Java 8 platform. The feature class combinations were set to the MaxEnt default, which automatically selects linear, quadratic, product, and hinge features according to the sample size of each species. The regularization multiplier (RM) was tuned by manually testing four values—0.5, 1.0, 1.5, and 2.0. For each species, the mean test AUC over 10 repeated random splits was initially used as the selection criterion. Although individual optimal values varied slightly during preliminary testing, we uniformly applied RM = 1.0 across all seven *Astilbe* species to ensure consistent model complexity and cross-species comparability. The performance metrics for all the species when RM = 1.0 are provided in **Supplementary Table 3**. For model validation, the occurrence records of each species were randomly partitioned into a training set (75%) and a test set (25%). This random partitioning and the subsequent modelling–evaluation procedure were independently repeated 10 times, and the optimal values across the 10 replicates were reported as the final AUC and TSS. All the MaxEnt models for the seven *Astilbe* species were calibrated using the entire terrestrial area within the national boundary of China as the background region, from which 10,000 background points were randomly generated. The maximum number of iterations was set to 500 (Phillips et al., 2006; Shi et al., 2023), and the convergence threshold was set to 10^-5^ (Jarnevich and Young, 2015). The jackknife test was used to assess variable importance, and model outputs were generated in logistic format, yielding continuous habitat suitability values ranging from 0 to 1. An initial MaxEnt model was run with all 23 candidate environmental variables to obtain the percent contribution and permutation importance for each predictor. Independently, pearson correlation coefficients (PCCs) were calculated for each species using IBM SPSS Statistics 26 on the basis of values extracted at the occurrence points, and a correlation matrix was generated (**Supplementary Figure 1**). Variable pairs with |r| > 0.8 were considered highly collinear (Cotrina et al., 2021); for each such pair, the variable with a lower percent contribution in the full-variable MaxEnt run was discarded. From the remaining set, variables with a percent contribution of exactly zero were further removed, as they provided no explanatory power. This two-step, noniterative screening procedure was applied independently to each species. The reduced predictor sets used in the final MaxEnt models are listed in **Supplementary Table 3**.

Model performance was evaluated using the area under the curve (AUC) and the true skill statistic (TSS). AUC values range from 0 to 1 and are insensitive to class imbalance (Parodi et al., 2022); higher values indicate stronger discrimination between presence and background points (Ma et al., 2021; Li et al., 2025). In accordance with Lian et al. (2024), models with AUCs ≥ 0.7 were considered acceptable for predicting potential species habitats. Recognizing that the AUC alone has inherent limitations (Guo et al., 2020), we additionally computed the TSS as a complementary metric. The TSS ranges from −1 to +1, with values of 0 or below indicating that the performance no better than random, and +1 representing perfect agreement (Allouche et al., 2006). The TSS was calculated using R Studio Desktop (AGPL v3). For each species, the model with the highest combined AUC and TSS among the 10 replicates that met the criterion of an AUC > 0.7 was selected. Under both the current and future climate scenarios, the training AUC values for all seven *Astilbe* species exceeded 0.85, and the TSS values were greater than 0.75. These values were substantially above the random expectation of 0.5, indicating that the models adequately captured the relationships between species occurrence and environmental predictors (**Supplementary Table 4**).

MaxEnt model outputs were processed in ArcGIS 10.8 (Esri, Redlands, CA, USA). For each species and each time period (2010s, 2030s, 2050s, and 2070s), the maximum training sensitivity plus specificity threshold (MaxSS), which was calculated from the current-period training data, was first applied to separate unsuitable areas from suitable areas (Liu et al., 2013). Within the suitable area, three subcategories were then delineated using the equal-interval method: low suitability, medium suitability, and high suitability. As a result, habitat suitability was classified into four final categories: unsuitable, low suitability, medium suitability, and high suitability. The numerical ranges defining each category are summarized in **Supplementary Table 5**. To avoid area distortion due to latitude, the suitability rasters were projected to the Albers equal-area conic projection for China (Central_Meridian: 105°, Standard_Parallel_1: 25°N, Standard_Parallel_2: 47°N, Latitude_Of_Origin: 0°). The area of each suitability class was then calculated using the SDM Toolbox (Brown and Anderson, 2014) by multiplying the cell count ([COUNT] in the attribute table) by the corresponding cell area (cell size × cell size). The total suitable area (sum of the low-, medium-, and high-suitability classes) was subsequently derived. The spatiotemporal dynamics of habitat suitability were characterized by determining the geographic centroid of suitable habitat for each species and period using the “Mean Center” tool. The direction and distance of centroid displacement between consecutive periods were measured with the “Points to Line” tool, which together represent the direction and magnitude of habitat shift. All spatial analyses were performed in ArcGIS (Liu et al., 2025).

## 3 Results

### 3.1 Key environmental factors affecting the distribution of *Astilbe*

**Figure 2:**
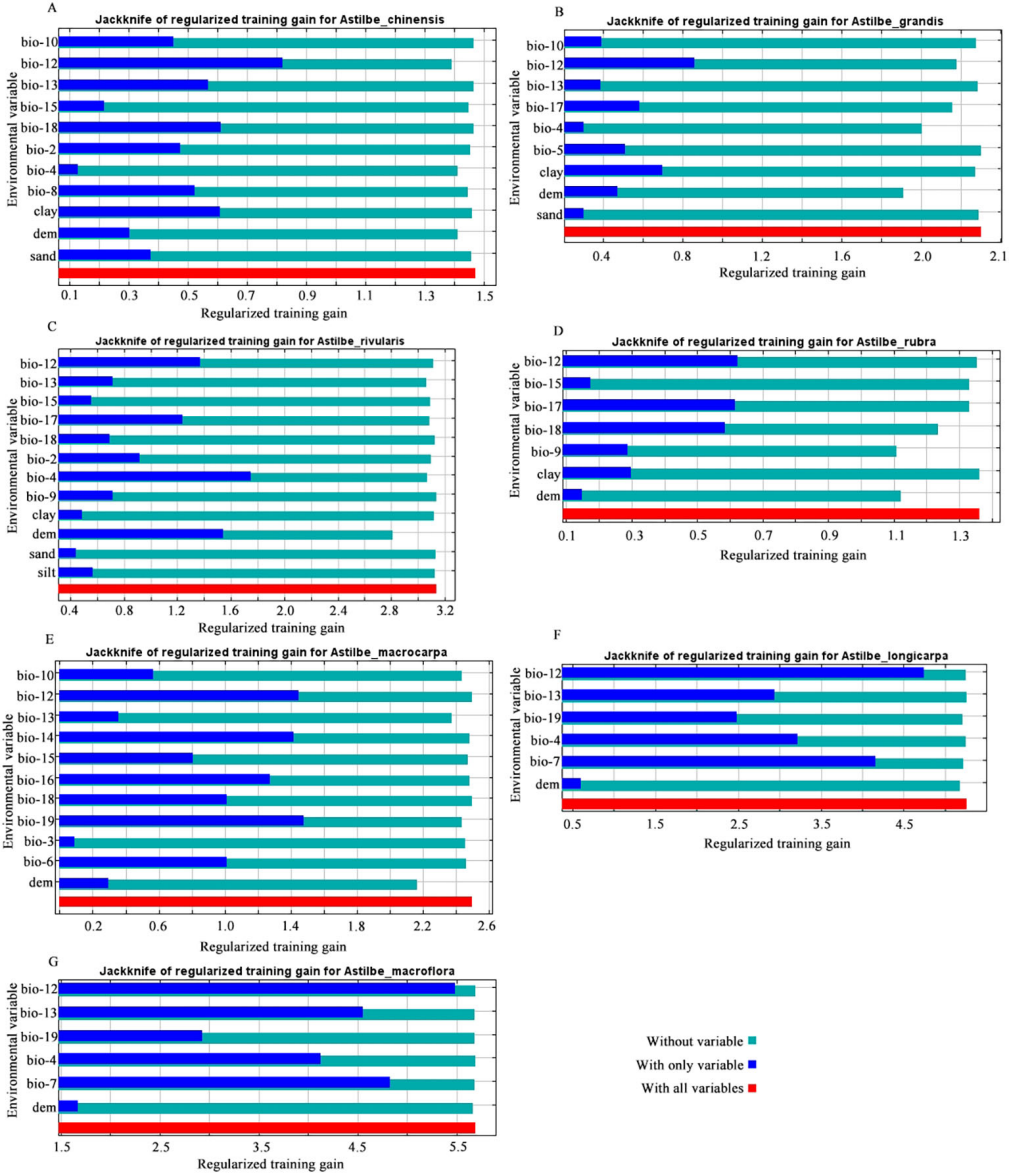
Jackknife analysis of regularized training gains for the genus *Astilbe*. The panel labels correspond to the following species: **(A)** *A. chinensis*, **(B)** *A. grandis*, **(C)** *A. rivularis*, **(D)** *A. rubra*, **(E)** *A. macrocarpa*, **(F)** *A. longicarpa*, and **(G)** *A. macroflora*.

**Figure 3:**
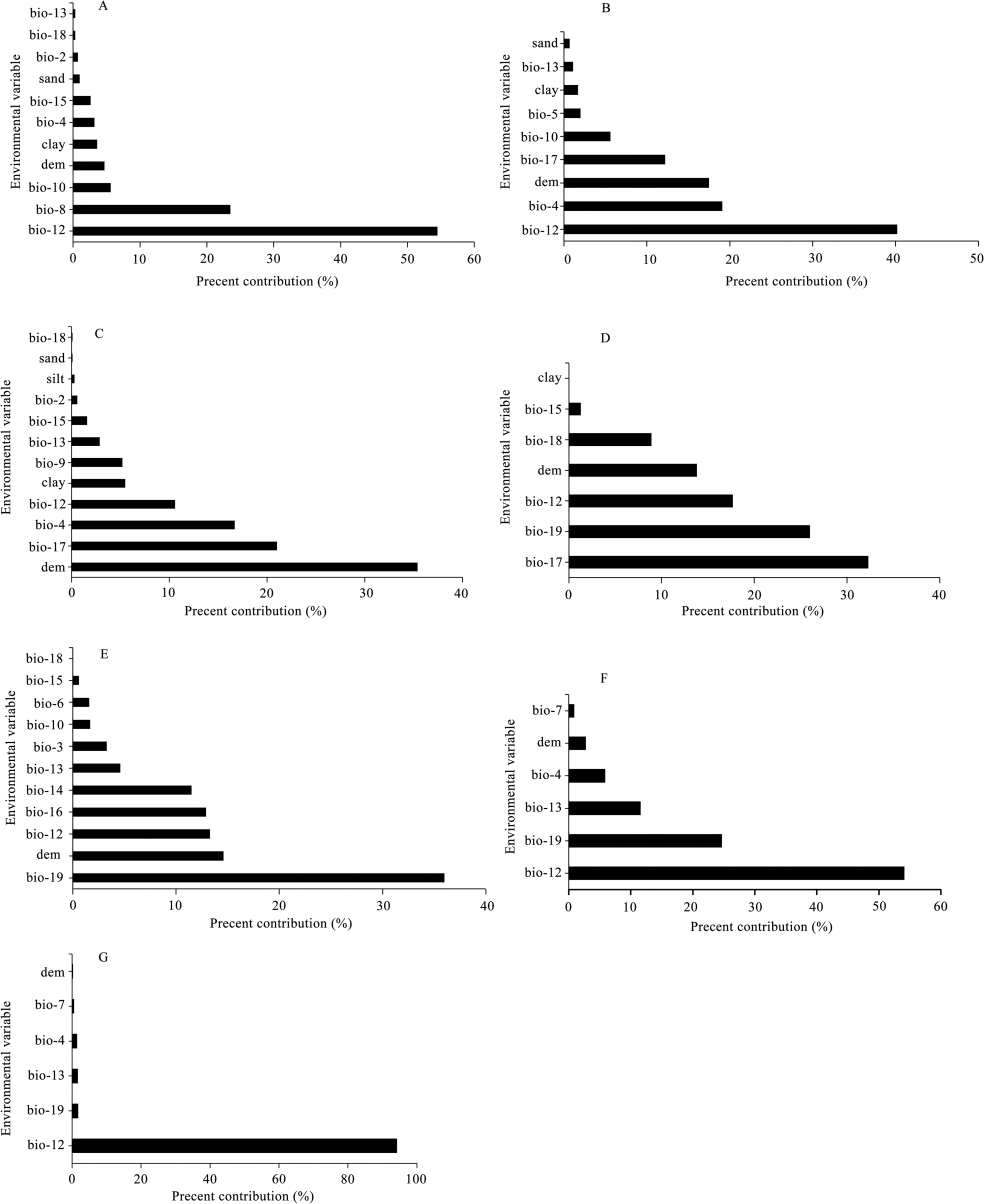
Percentage contribution of each environmental variable to predicting suitable habitats for the genus *Astilbe* in China. The panel labels correspond to the following species: **(A)** *A. chinensis*, **(B)** *A. grandis*, **(C)** *A. rivularis*, **(D)** *A. rubra*, **(E)** *A. macrocarpa*, **(F)** *A. longicarpa*, and **(G)** *A. macroflora*.

To identify the environmental variables most strongly associated with the biogeographic patterns of *Astilbe*, we used three independent lines of evidence to determine the key environmental factors. First, we examined the response curve of each predictor to assess whether it exhibited aberrant shapes inconsistent with the species’ known ecological characteristics. Second, we screened variables by cumulative percent contribution and retained those that together accounted for more than 85% of the variance (Huang et al., 2024) (**Figures 2**, **3**). Third, we inspected the jackknife test of regularized training gain: the variable with the highest gain when used alone was interpreted as containing the most information by itself, whereas the variable whose removal caused the greatest decrease in gain was considered to carry unique information not shared by other predictors.

Based on this synthesis, the following variables emerged as the most influential for each species: *A. chinensis*—bio12, bio8, bio10, and DEM; *A. grandis*—bio12, bio4, DEM, bio17; *A. rivularis*—DEM, bio17, bio4, bio12; *A. rubra*—bio17, bio9, bio12, and DEM; *A. macrocarpa*—bio19, DEM, bio12, bio16, and bio14; *A. longicarpa*—bio12, bio19, bio13, and DEM; and *A. macroflora*—bio12 and DEM. Across all species, annual precipitation (bio12) and DEM were the variables most frequently identified as important. Response curves illustrating the relationships between these key environmental variables and predicted habitat suitability are provided in **Supplementary Figure 2**.

### 3.2 Prediction of habitat suitability prediction

#### 3.2.1 Current climate scenario prediction

**Figure 4:**
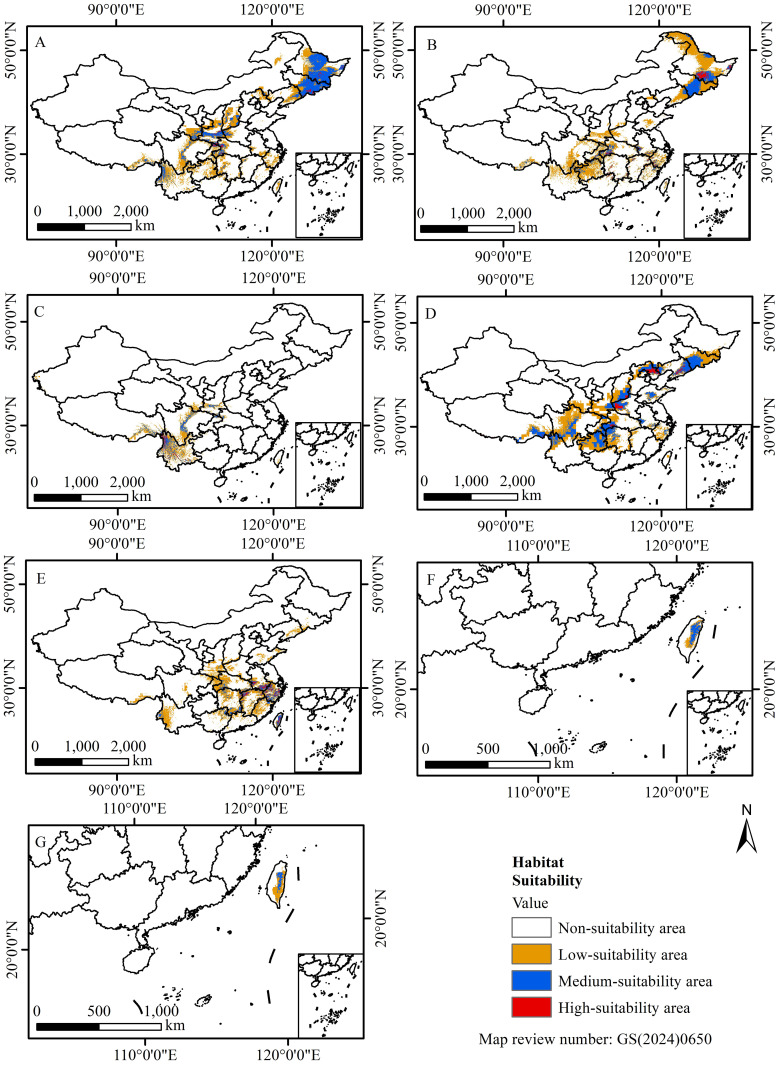
Distribution patterns of suitable habitats for *Astilbe* under current climate conditions in the 2010s. The panel labels correspond to the following species: **(A)** *A. chinensis*, **(B)** *A. grandis*, **(C)** *A. rivularis*, **(D)** *A. rubra*, **(E)** *A. macrocarpa*, **(F)** *A. longicarpa*, and **(G)** *A. macroflora*.

As shown in **Figure 4**, the potential suitable habitats of the seven *Astilbe* species predicted by MaxEnt were largely consistent with their recorded distributions. Under the current climate, *A. rubra* had the greatest suitable habitat area, accounting for approximately 15.60% of the study area and spanning Southwest, Northwest, North, and Northeast China. *A. chinensis* came next, representing approximately 14.04% of the study area, and occurred mainly in Northeast, Northwest, and Southwest China. The suitable habitat of *A. grandis* was concentrated in Northeast and Southwest China, whereas that of *A. rivularis* was highly concentrated in Southwest China. The ranges of *A. longicarpa* and *A. macroflora* were the most restricted, occupying only approximately 0.14% and 0.15% of the study area, respectively, and were largely confined to Taiwan Province, East China; notably, no highly suitable areas were identified for either species under the current climate. Additionally, the suitable habitats of *A. chinensis*, *A. grandis*, and *A. rubra* showed pronounced geographic overlap in Northeast China and Southwest China (**Figure 4**, **Table 2**).

**Table 2:** Area distribution of suitable zones for *Astilbe* at various levels under different climate scenarios over the years (× 10^4^km^2^).

Species	Scenarios	Low-suitability area	Medium-suitability area	High-suitability area	Total-suitability area
*A. chinensis*	2010s	86.92(9.06%)	45.83(4.78%)	1.95(0.20%)	134.71(14.04%)
	2030s	85.10(8.87%)	83.56(8.71%)	20.69(2.16%)	189.35(19.73%)
	2050s	86.04(8.96%)	79.82(8.32%)	20.54(2.14%)	186.41(19.42%)
	2070s	88.02(9.17%)	82.01(8.54%)	19.38(2.02%)	189.41(19.73%)
*A. grandis*	2010s	93.88(9.78%)	21.93(2.28%)	3.92(0.41%)	119.72(12.47%)
	2030s	83.70(8.72%)	18.87(1.97%)	9.09(0.95%)	111.67(11.63%)
	2050s	89.23(9.30%)	21.77(2.27%)	9.13(0.95%)	120.13(12.52%)
	2070s	92.63(9.65%)	23.67(2.47%)	10.83(1.13%)	127.13(13.25%)
*A. rivularis*	2010s	29.61(3.08%)	10.41(1.08%)	1.52(0.16%)	41.54(4.33%)
	2030s	30.96(3.23%)	11.26(1.17%)	4.09(0.43%)	46.30(4.82%)
	2050s	30.11(3.14%)	11.64(1.21%)	4.48(0.47%)	46.23(4.82%)
	2070s	29.14(3.04%)	11.09(1.16%)	3.84(0.40%)	44.07(4.59%)
*A. rubra*	2010s	96.69(10.07%)	48.90(5.10%)	4.17(0.43%)	149.76(15.60%)
	2030s	62.28(6.49%)	64.71(6.74%)	27.23(2.84%)	154.23(16.07%)
	2050s	65.46(6.82%)	64.30(6.70%)	22.34(2.33%)	152.09(15.85%)
	2070s	67.65(7.05%)	63.62(6.63%)	25.36(2.64%)	156.63(16.32%)
*A. macrocarpa*	2010s	64.79(6.75%)	13.99(1.46%)	3.61(0.38%)	82.39(8.58%)
	2030s	96.49(10.05%)	56.75(5.91%)	29.10(3.03%)	182.34(19.00%)
	2050s	105.94(11.04%)	59.36(6.18%)	31.77(3.31%)	197.07(20.53%)
	2070s	107.24(11.17%)	61.37(6.39%)	39.78(4.14%)	208.39(21.71%)
*A. longicarpa*	2010s	0.66(0.07%)	0.67(0.07%)	0.00(0.00%)	1.33(0.14%)
	2030s	0.30(0.03%)	0.29(0.03%)	0.56(0.06%)	1.15(0.12%)
	2050s	0.30(0.03%)	0.28(0.03%)	0.55(0.06%)	1.14(0.12%)
	2070s	0.30(0.03%)	0.29(0.03%)	0.56(0.06%)	1.15(0.12%)
*A. macroflora*	2010s	1.05(0.11%)	0.39(0.04%)	0.00(0.00%)	1.45(0.15%)
	2030s	1.03(0.11%)	0.63(0.07%)	0.54(0.06%)	2.20(0.23%)
	2050s	0.98(0.10%)	0.65(0.07%)	0.58(0.06%)	2.21(0.23%)
	2070s	1.00(0.10%)	0.65(0.07%)	0.56(0.06%)	2.21(0.23%)

#### 3.2.2 Future climate scenario predictions

Future dynamics of suitable habitat for *Astilbe* species from the 2030s to the 2070s were projected under the SSP1−2.6 scenario. Under this scenario, China experiences an overall increase in both temperature and precipitation over the period 2000–2080 (**Supplementary Figure 3**). The results indicated that, except for *A. longicarpa*, the total suitable habitat area of the other six species was projected to expand.

**Figure 5:**
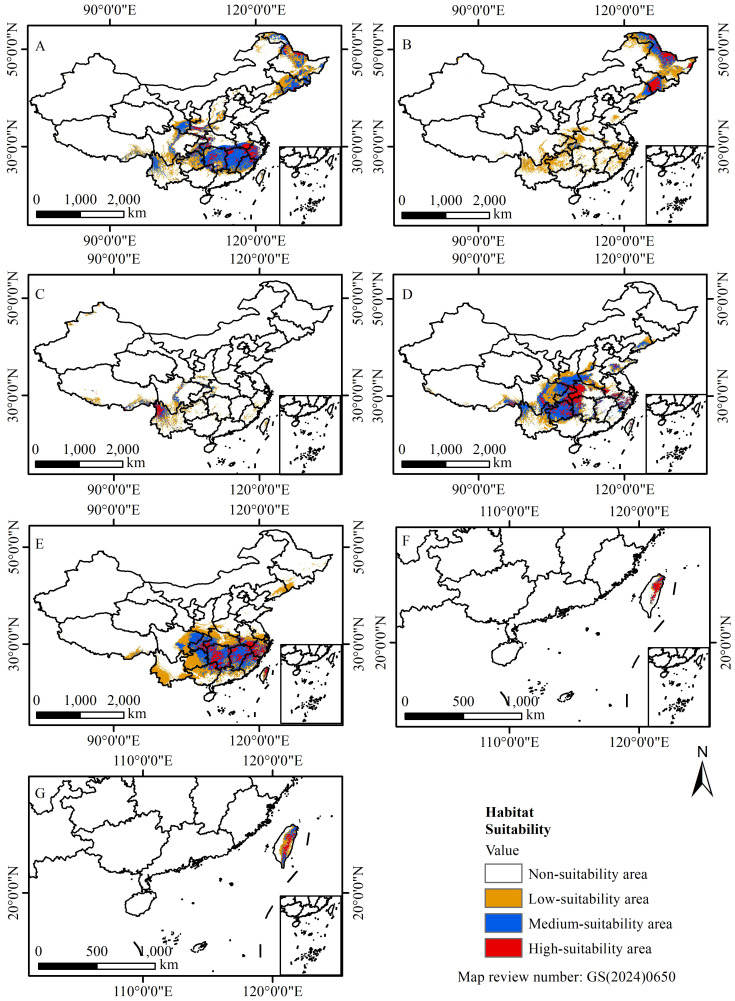
Distribution patterns of suitable habitats for *Astilbe* under future climate conditions in the 2030s. The panel labels correspond to the following species: **(A)** *A. chinensis*, **(B)** *A. grandis*, **(C)** *A. rivularis*, **(D)** *A. rubra*, **(E)** *A. macrocarpa*, **(F)** *A. longicarpa* and **(G)** *A. macroflora*.

**Figure 6:**
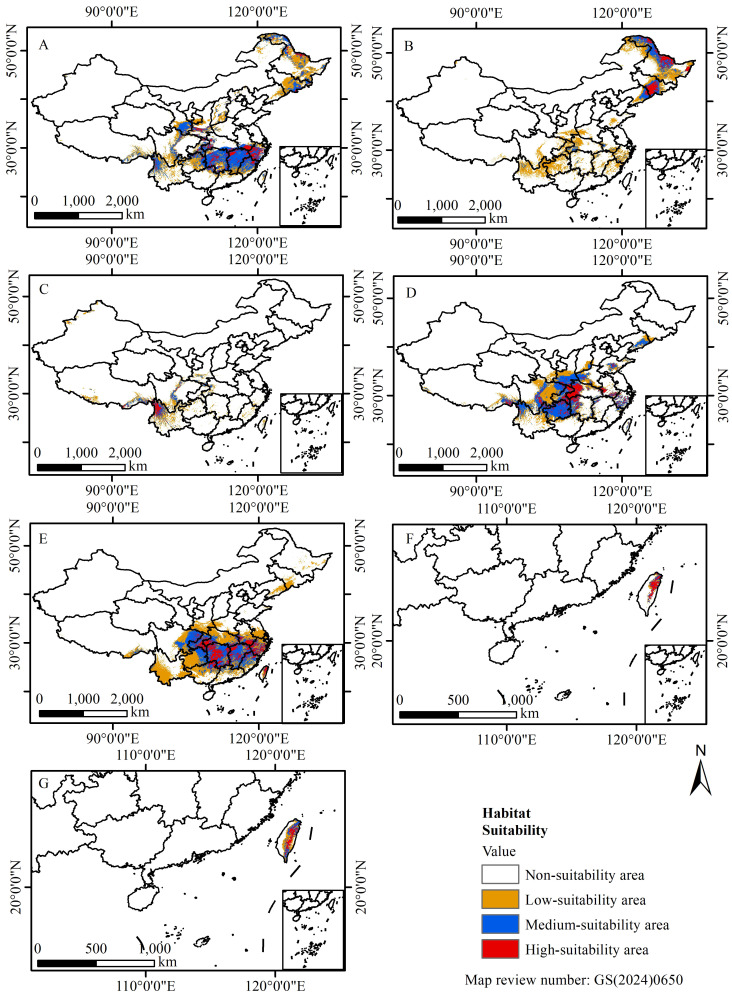
Distribution patterns of suitable habitats for *Astilbe* under future climate conditions in the 2050s. The panel labels correspond to the following species: **(A)** *A. chinensis*, **(B)** *A. grandis*, **(C)** *A. rivularis*, **(D)** *A. rubra*, **(E)** *A. macrocarpa*, **(F)** *A. longicarpa* and **(G)** *A. macroflora*.

**Figure 7:**
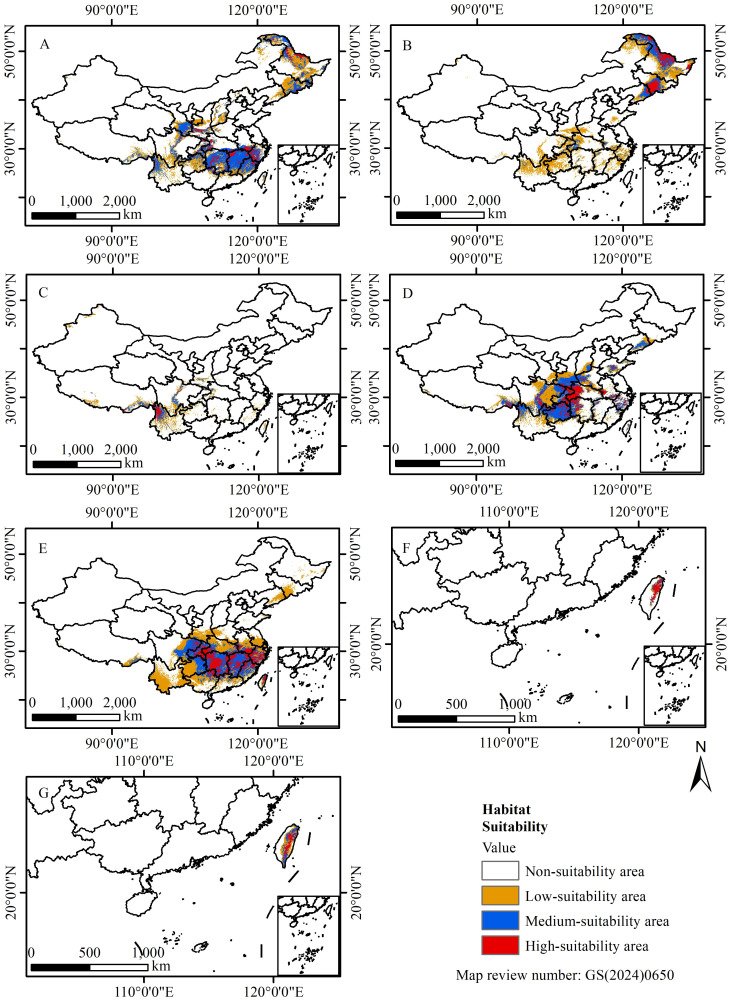
Distribution patterns of suitable habitats for *Astilbe* under future climate conditions in the 2070s. The panel labels correspond to the following species: **(A)** *A. chinensis*, **(B)** *A. grandis*, **(C)** *A. rivularis*, **(D)** *A. rubra*, **(E)** *A. macrocarpa*, **(F)** *A. longicarpa* and **(G)** *A. macroflora*.

The spatial configuration of suitable habitat shifted substantially for *A. chinensis*, *A. rubra*, and *A. macrocarpa* (**Figures 5**–**7**; **Table 3**). Specifically, the highly suitable areas for *A. chinensis* shifted from Northeast and Central China towards Northeast, Central, and East China, with the largest share occurring in East China. Its moderately suitable areas moved from Northeast China towards Central and East China, contributing to an overall expansion of its suitable range. With respect to *A. rubra*, the distribution of suitable habitat changed from a relatively dispersed pattern to a pattern concentrated in Southwest, Northwest, and Central China. *A. macrocarpa* exhibited a particularly pronounced expansion: newly emerged medium− and high−suitability areas were concentrated in Central and East China, and its spatial pattern became increasingly aggregated.

**Table 3:** Centroid coordinates and migration distance of *Astilbe*.

Species	Period	Longitude(°E)	Latitude(°N)	Migration distance(~ km)
*A. chinensis*	2010s	114.018641	34.971598	–
	2030s	113.22082	33.65733	164.98
	2050s	112.662576	33.27501	66.81
	2070s	113.190234	33.881326	83.66
*A. grandis*	2010s	114.18812	32.948798	–
	2030s	117.856251	37.60927	619.97
	2050s	117.285835	36.513056	133.55
	2070s	117.253768	36.560657	6.07
*A. rivularis*	2010s	101.668379	27.750648	–
	2030s	103.07903	28.234303	147.89
	2050s	102.821431	28.193734	25.48
	2070s	103.002418	28.196141	17.62
*A. rubra*	2010s	109.633416	31.643417	–
	2030s	110.198856	29.592533	236.49
	2050s	110.352472	29.896782	37.14
	2070s	110.411992	29.948809	8.14
*A. macrocarpa*	2010s	114.573654	29.378869	–
	2030s	111.610839	29.573396	285.03
	2050s	112.702138	29.337788	107.96
	2070s	112.603405	29.297962	10.49
*A. longicarpa*	2010s	121.264641	23.953185	–
	2030s	121.236744	23.881678	8.39
	2050s	121.257932	23.981894	11.27
	2070s	121.276109	24.02986	5.61
*A. macroflora*	2010s	121.089444	23.502988	–
	2030s	121.16355	23.801573	33.80
	2050s	121.209399	23.790923	4.83
	2070s	121.194186	23.79879	1.78

The values in parentheses indicate the proportion (%) of the total study area.

### 3.3 Response of suitable areas to climate change

**Figure 8:**
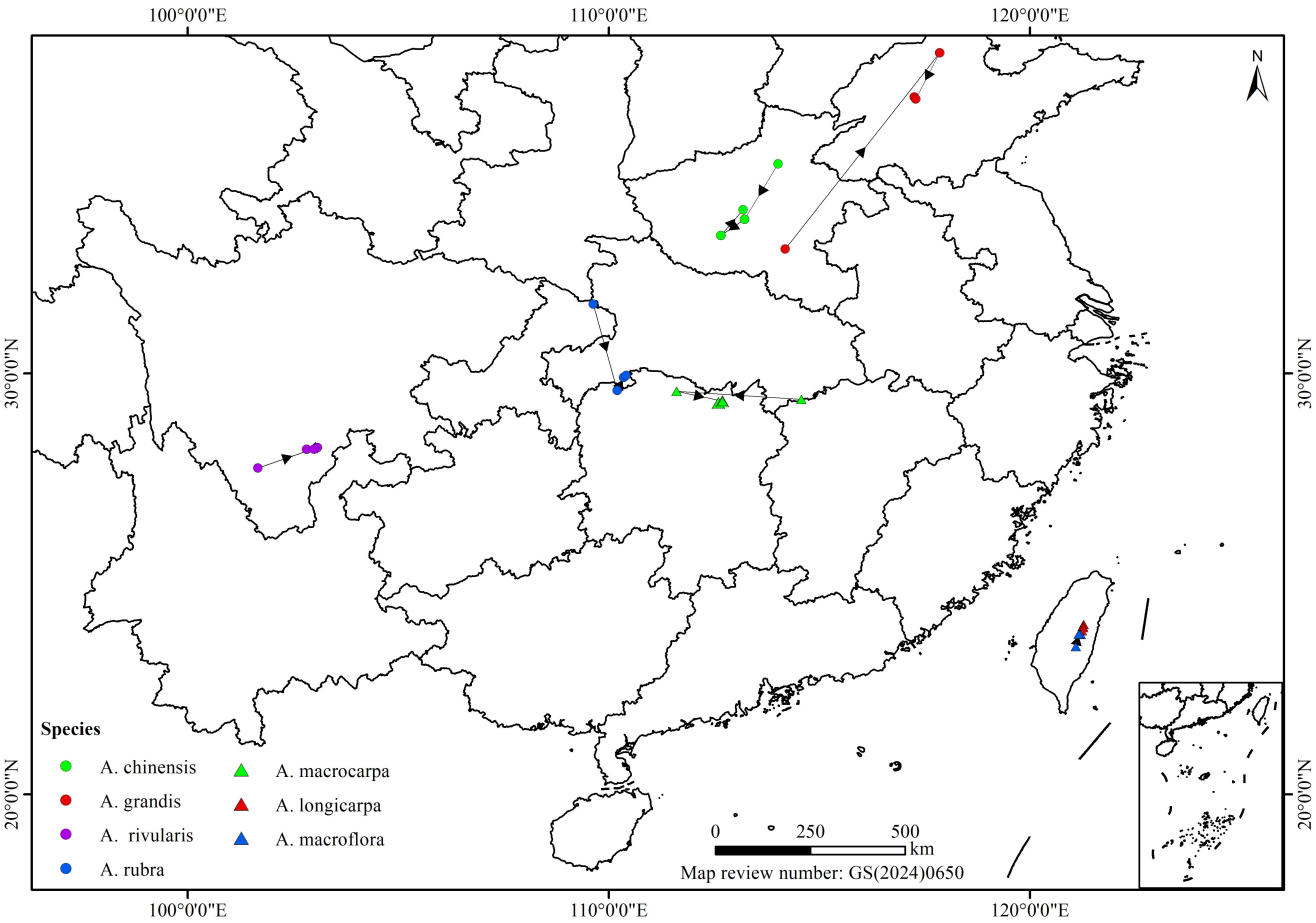
Centroid locations of suitable habitats for *Astilbe* under different climate scenarios.

Centroid analysis revealed divergent latitudinal and longitudinal displacement patterns of suitable habitat across the seven *Astilbe* species from the 2010s to the 2070s. The centroids of *A. grandis*, *A. rivularis*, *A. longicarpa*, and *A. macroflora* shifted towards higher latitudes, whereas those of *A. chinensis*, *A. rubra*, and *A. macrocarpa* moved towards lower latitudes—with *A. macrocarpa* also displaying a predominantly westwards displacement. Among these, the northwards shift was most pronounced for *A. grandis* within mainland China, spanning approximately 3.61° in latitude (from approximately 32.95° N to 36.56° N), with the greatest displacement occurring between the 2010s and the 2030s (approximately 619.97 km). The centroid of *A. rubra* shifted southwards by approximately 2.35°, with a maximum displacement of approximately 236.49 km. *A. macrocarpa* exhibited a westwards centroid displacement of approximately 1.97° in longitude, with a maximum displacement of approximately 285.03 km. The centroid displacements of the remaining species (*A. chinensis*, *A. rivularis*, *A. macroflora*, and *A. longicarpa*) progressively decreased (**Figure 8**, **Table 3**).

This study was conducted under SSP1−2.6, a low-emission scenario, to systematically project the direction and magnitude of suitable habitat shifts for *Astilbe* species under climate change. Plant distributions are jointly constrained by climatic, edaphic, and topographic factors. The integrated projections indicate that, during 2041–2080, climate change in China is likely to generate new suitable areas and promote the spatial expansion of climatically suitable habitats, potentially providing favorable climatic conditions for several *Astilbe* species. However, a declining trend in total suitable area was projected for *A. longicarpa*. Notably, the spatial pattern of suitable habitat for *A. chinensis* has markedly departs from its current configuration: the centroids of its highly and moderately suitable areas have shifted from Northeast China towards Central and East China, indicating a projected southwards expansion of its climatically suitable range.

**Figure 9:**
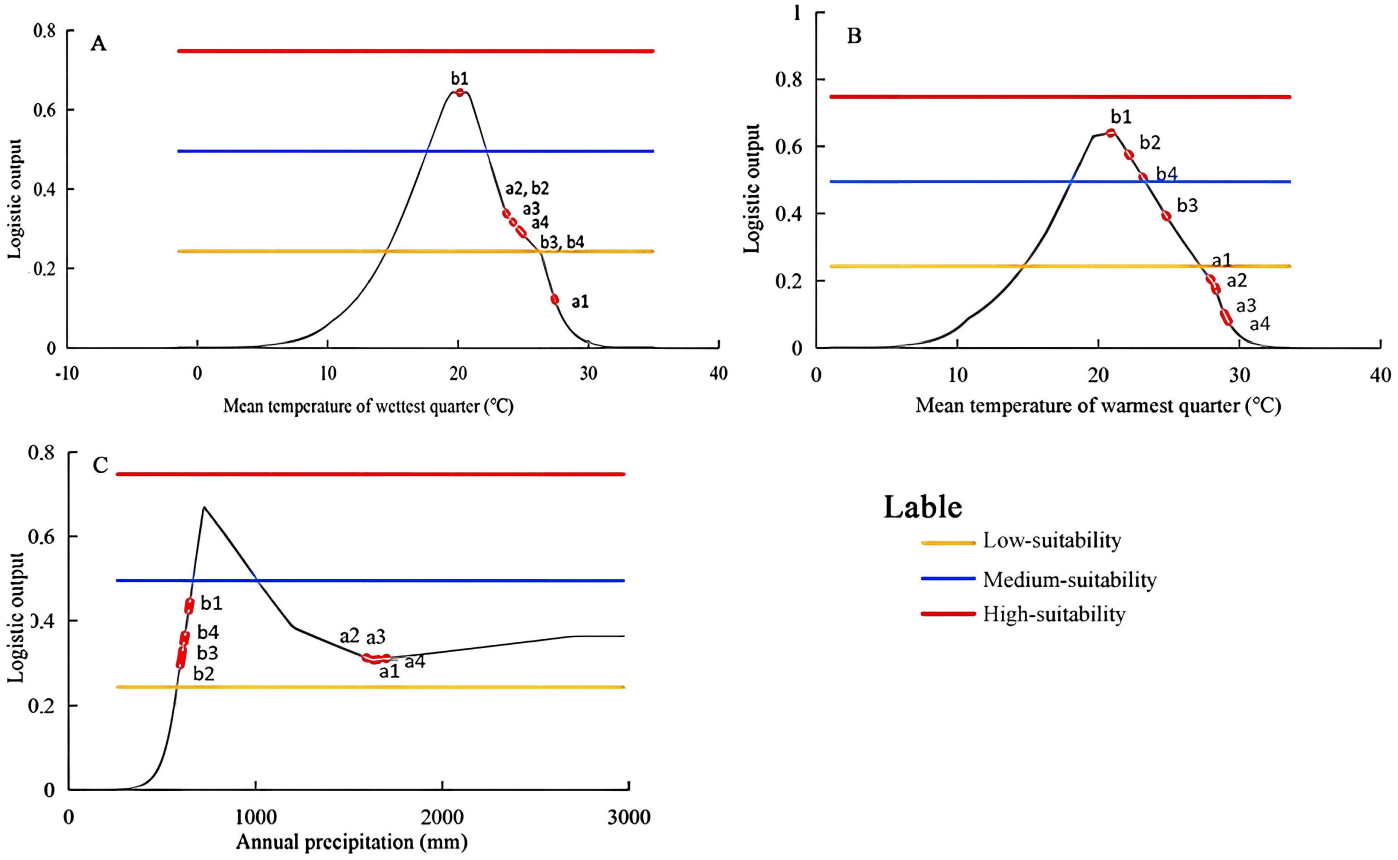
Niche distribution of key environmental factors in the suitable habitat of *A. chinensis*. panels **(A–C)** show bio8, bio10, and bio12, respectively. Within each panel, points labeled “a” denote the average over Hunan, Jiangxi, Zhejiang, and Fujian provinces; points labeled “b” denote the average over Jilin and Heilongjiang provinces. The numerical suffixes 1–4 following the letters indicate the 2010s, 2030s, 2050s, and 2070s, respectively. Temperature is expressed in °C; precipitation is in mm.

To further examine the environmental correlates of these shifts in *A. chinensis*, response curves derived from the MaxEnt model were analyzed separately for the temperature and precipitation conditions in its southern and northern suitable ranges (**Figure 9**). Within its primary suitable range, combinations of relatively stable mean temperature of the coldest quarter (bio10) and annual precipitation (bio12), together with a lower mean temperature of the warmest quarter (bio8), were associated with higher predicted habitat suitability. In contrast, elevated bio8 and bio10 under relatively stable bio12 were associated with a reduction in predicted suitability.

## 4 Discussion

### 4.1 The impacts of environmental variables on the potential distribution of *Astilbe*

The potential distribution of a species is shaped by multiple environmental factors and reflects statistical associations between its habitat preferences and climatic conditions (Sanczuk et al., 2022). Temperature and precipitation are widely recognized as the primary determinants of species ranges (Coelho et al., 2023), and suitable hydrothermal conditions are often critical for plant growth and survival (Li et al., 2025). In this study, we applied the MaxEnt model to evaluate the species–environment relationships and habitat suitability for seven *Astilbe* species. As shown in **Figure 3**, annual precipitation (bio12) and DEM were the most influential variables, followed by precipitation during the wettest month (bio13), temperature seasonality (bio4), precipitation seasonality (bio15), precipitation during the warmest quarter (bio18), and soil silt content. Over the 2000–2080 projection period, the spatial pattern of DEM remained essentially unchanged; as a result, climatic variables served as the dominant controls on distribution dynamics, while edaphic and topographic factors played secondary yet complementary roles in shaping the distribution patterns of *Astilbe* species. Previous studies have similarly demonstrated that topographic features—such as elevation, slope, and aspect—and soil properties strongly influence plant distributions (Tang et al., 2019; Xie et al., 2019; Luo et al., 2021). For instance, Wang et al. (2023) identified temperature seasonality, annual precipitation, and the DEM as the major drivers of the distribution of *Quercus sect. Heterobalanus* in China, and Que and Dai (2022) reported that *A. chinensis* is broadly adapted to a range of soil textures and occurs primarily in valleys, forest edges, shrublands, and hilly meadows. Overall, although edaphic and topographic factors were identified as secondary variables in this study, their contribution to the distribution patterns of *Astilbe* remains ecologically important.

In this study, the DEM contributed substantially to the MaxEnt predictions for the genus *Astilbe*, which is consistent with the finding of Que and Dai (2022) that the annual precipitation and precipitation of the warmest quarter are associated with the distribution of *A. chinensis*. However, the habitat suitability predictions for *A. longicarpa* and *A. macroflora* showed minimal sensitivity to the DEM (contribution rates below 3% for both species) and were instead dominated by climatic variables. This pattern coincides spatially with the pronounced topographic discontinuity across the Taiwan Strait, where extensive low−elevation corridors—common on the mainland—are largely absent. One possible interpretation is that this elevational contrast limits the availability of suitable climatic conditions for these two species outside of Taiwan, although confirming this hypothesis would require independent evidence beyond distribution modelling. In addition, the potentially suitable habitats of *A. longicarpa* and *A. macroflora* each cover less than 3 × 10^4^ km². Given such restricted predicted ranges, these two species may face heightened extinction risk under future climate scenarios and therefore warrant prioritized conservation assessment.

*A. macrocarpa* is endemic to East and Central China, with records documented in Anhui, Zhejiang, Jiangxi, Fujian, Hunan, and Sichuan Provinces. The MaxEnt predictions obtained in this study suggest that its distribution is statistically associated with environmental variables representing extreme temperature and drought stress. The range of the species lies within a subtropical monsoon climate zone and coincides spatially with a complex mountainous terrain, where localized microenvironmental conditions may offer suitable habitats. These mountains coincide spatially with areas of increased environmental heterogeneity beyond the gradient captured by the model and, together with regional climate patterns, delineate relatively stable climatic–topographic zones. The climatic limitations linked to low temperature and drought may be associated with a reduced probability of occurrence in surrounding areas, contributing to the patchy distribution observed in natural populations.

### 4.2 Response of the potential *Astilbe* distribution to climate change

Under future climate warming, the overall trend from the 2010s to the 2070s indicates an expansion of the total suitable habitat area for all *Astilbe* species except *A. longicarpa*, albeit with minor fluctuations. Spatially, as temperature and precipitation increase, the redistribution of suitable habitat can be characterized by two concurrent trends: (1) in the core areas of certain species, zones initially classified as having low suitability tend to transition to medium suitability, and medium−suitability zones shift to high suitability, resulting in a pattern of increasing suitability concentrated within the most favorable areas (e.g., *A. grandis* and *A. rivularis*); (2) in some peripheral areas, zones previously projected as medium suitability are reclassified as low suitability in subsequent time periods, indicating a decline in predicted suitability at the range margins (e.g., *A. rubra*). Consequently, although individual species exhibit varying degrees of range contraction or expansion, the spatial dynamics of suitable habitats for *Astilbe* can be described as a dual pattern: an increase in suitability classes within core areas, coupled with fluctuations in suitability at range margins. At the genus level, the overall trend points to a simultaneous increase in the concentration of higher suitability classes within climatically optimal areas, along with a progressive expansion into newly suitable peripheral zones.

Climate change can drive the expansion or contraction of species ranges, thereby altering their geographic distribution patterns (Hu et al., 2024). Our results indicate that the centroids of the potentially suitable habitats of *A. grandis*, *A. rivularis*, *A. longicarpa*, and *A. macroflora* generally shifted towards higher latitudes over time. This spatial trend is consistent with documented shifts in the potential distributions of several plant taxa under global warming. For example, Qiao et al. (2025) predicted that the potential distribution centroids of certain *Litsea* species (e.g., *L. cubeba* and *L. rubescens*) would move towards higher latitudes, and Han et al. (2025) reported a northwards expansion trend in the potentially suitable habitats of *Sophora velutina*, *S. japonica*, and *Robinia pseudoacacia*. In contrast, the suitable habitat centroids of *A. chinensis*, *A. rubra*, and *A. macrocarpa* shifted towards lower latitudes, mirroring the pattern of contraction towards lower latitudes and lower elevations described by Shan et al. (2025). At the genus level, the spatial displacement of potentially suitable habitats for *Astilbe* species varied across successive time periods, resulting in complex, species-specific patterns that align with the findings of Li et al. (2024). Collectively, these findings reinforce the well-established ecological paradigm in which “species track climate change by shifting their ranges towards higher latitudes and elevations” (Šimala et al., 2023).

However, *A. chinensis* is currently also widely distributed in the cold regions of Northeast China. The observed shift in its suitable habitat centroid towards lower latitudes and the southwards expansion of its suitable range diverge from the more commonly reported poleward shifts under climate warming. Osmolovsky et al. (2025) proposed that species that shift towards lower latitudes or the equator may not exhibit an active adaptive strategy to elevated temperatures, but rather respond to climate-mediated changes in interspecific interactions. This perspective offers a plausible ecological context for the counterintuitive spatial trend observed in *A. chinensis* and suggests that the distribution dynamics of this genus may be influenced by climate−driven alterations in biotic interactions. Our findings align with recent SDM studies on medicinal plants in East Asia, which commonly document poleward and upslope shifts in species ranges under climate warming. For instance, Zhong et al. (2025) projected a net 28 % loss of suitable habitat for *Paris polyphylla* var. *yunnanensis* by 2070, primarily driven by drought stress—a pattern that resonates with the sensitivity of *A. macrocarpa* to extreme precipitation observed in our study. Similarly, Zuo et al. (2023) identified elevation and temperature seasonality as the dominant factors structuring the distribution of *Dendrobium nobile*, further underscoring the role of climatic stability in shaping the distributions of montane herbaceous species across the region. Given the medicinal value of *Astilbe* rhizomes, dynamically updating conservation strategies based on habitat suitability projections merits consideration. For critically restricted endemics such as *A. longicarpa*, priority should be given to germplasm banking and cross-jurisdictional conservation coordination to prevent the irreversible loss of genetic diversity.

Several limitations of this study should be acknowledged. The model incorporated only bioclimatic variables, soil properties, and topographic factors as predictors, and therefore did not account for critical ecological processes such as dispersal limitation, interspecific interactions (e.g., competition or facilitation), or anthropogenic disturbances (Soberon and Peterson, 2005; Barve et al., 2011). This simplification constrains the ecological realism of the projections, particularly in human-modified landscapes where nonclimatic drivers may override climate signals; as an initial effort to characterize climate-driven habitat shifts in *Astilbe*, this work provides a preliminary baseline that can be refined through the inclusion of dynamic land cover data, human footprint metrics, and community-level biotic interactions in future modelling. In terms of model evaluation, the current assessment relies primarily on the AUC and TSS derived from repeated random subsampling, with hyperparameter optimization limited to manual tuning of the regularization multiplier. Although the AUC, as a threshold-independent metric, effectively reflects a model’s ability to distinguish presence from background points, it does not fully capture classification performance at specific thresholds, and future studies could build on this framework by incorporating threshold−dependent metrics such as Kappa alongside the AUC and TSS to enable a more comprehensive assessment of model performance. A further constraint is that this study was restricted to a single climate model (ACCESS-CM2) and a single emission scenario (SSP1-2.6). This design was deliberately chosen not to generate multimodel ensemble projections, but rather to characterize the lower bound of potential habitat impacts under the most optimistic mitigation pathway. SSP1-2.6 represents a strong emission reduction scenario that is compatible with the Paris Agreement’s 1.5–2 °C target; if significant habitat loss or range displacement is projected even under this minimal warming trajectory, the impacts under higher emission scenarios are almost certainly more severe. The results should therefore be interpreted as a conservative baseline assessment, identifying the minimum unavoidable impacts that remain even under ambitious global decarbonization. A key limitation of this single-scenario approach is that it cannot capture the structural uncertainty inherent in future climate projections, nor can it quantify the full range of species responses under alternative emission pathways. These constraints limit the generalizability of the findings beyond the defined scenario, and future studies incorporating multiple climate models and emission pathways are needed to bracket the potential range of outcomes and strengthen the reference value of such projections for conservation planning.

Given the escalating pressures from overharvesting and native habitat loss driven largely by the growing demand for traditional Chinese medicinal materials (Zhou et al., 2022)—, the projected shifts in habitat suitability offer spatially explicit information that can guide conservation priority-setting. Our results indicate that both the current native ranges of *Astilbe* species and the emerging high-suitability areas identified in this study warrant attention in future conservation assessments. Species distributed across coastal, inland, and insular regions of China are exposed to divergent climate trajectories and fine-scale habitat heterogeneity; however, the degree to which these projected changes in suitability translate into realized range shifts depends on factors beyond the scope of this study, including dispersal capacity, land-use change, and biotic interactions. Among the seven taxa examined, *A. longicarpa* and *A. macroflora* were identified as the species with the greatest potential vulnerability, because of their extremely restricted projected suitable areas (each < 3 × 10^4^ km²) and the absence of highly suitable habitat under current conditions. For such range−restricted species, field-based population surveys and genetic assessments are needed to determine whether *in situ* protection alone is sufficient or whether complementary measures—such as *ex situ* conservation and population reinforcement—should be considered. The spatial outputs of this study provide an initial framework for guiding such targeted investigations and for prioritizing regions where conservation resources may be most effectively deployed.

## 5 Conclusions

In this study MaxEnt modelling, was used to integrate field survey data, herbarium records, and 23 environmental variables to project the potential distribution and habitat suitability dynamics of seven *Astilbe* species under climate change from 2000 to 2080. The results revealed that annual precipitation (bio12) and (DEM had the strongest statistical associations with the geographic distribution of the genus, while temperature and soil factors played secondary roles. Under current climatic conditions, the core suitable areas are concentrated mainly in Northeast and Southwest China, with pronounced spatial differentiation among species, reflecting niche differences in niches; *A. rubra* had the greatest predicted suitable habitat area, whereas *A. longicarpa* had the smallest.

Under future climate scenarios, the total suitable habitat area of all species except *A. longicarpa* was projected to expand. From the 2010s to the 2070s, the direction of centroid displacement for suitable habitats varied across species and time periods. Several species exhibited an initial northwards shift followed by a southwards shift, although the overall trajectory remained predominantly northwards. At the genus level, the spatial redistribution of suitable habitat was characterized by species-specific and directionally heterogeneous patterns.

This study identifies statistical associations between shifting hydrothermal gradients and the spatial redistribution of potentially suitable habitats for *Astilbe* species, underscoring the need for heightened conservation attention towards range-restricted taxa. For species with elevated potential vulnerability, such as *A. longicarpa* and *A. macroflora*, population monitoring should be intensified within existing native ranges, and the feasibility of *ex situ* conservation and germplasm preservation should be assessed considering projected future suitable areas. The spatial predictions generated in this study provide a preliminary framework to guide such targeted conservation assessments.

These findings offer spatially explicit information to inform conservation planning for medicinal plant resources under climate change and serve as a reference for the sustainable utilization of *Astilbe* germplasm.

## Data Availability

The original contributions presented in the study are included in the article/**Supplementary Material**. Further inquiries can be directed to the corresponding authors.
