# Supplementary material for: Projected shifts in the potential distribution of Astilbe under the SSP1-2.6 climate scenario
Source: Front Plant Sci. 2026 Jul 16;17:1887603. doi: 10.3389/fpls.2026.1887603 (PMC13422459; doi:10.3389/fpls.2026.1887603)
Supplement: Supplementary file 1 [file DataSheet1.docx]

Supplementary Material

# Supplementary Figures


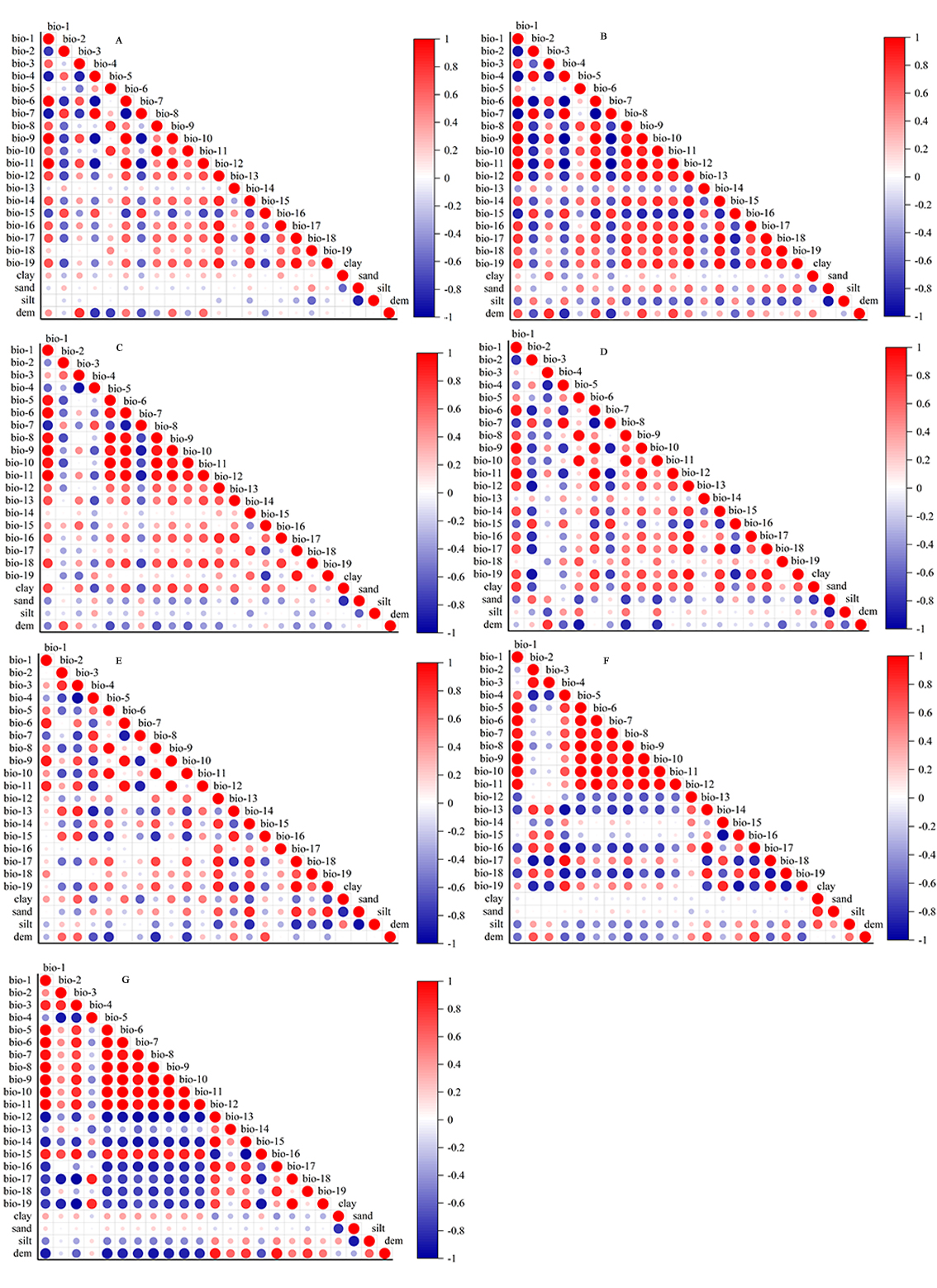


Supplementary Figure 1 Correlation plot of environmental factors for *Astilbe*.


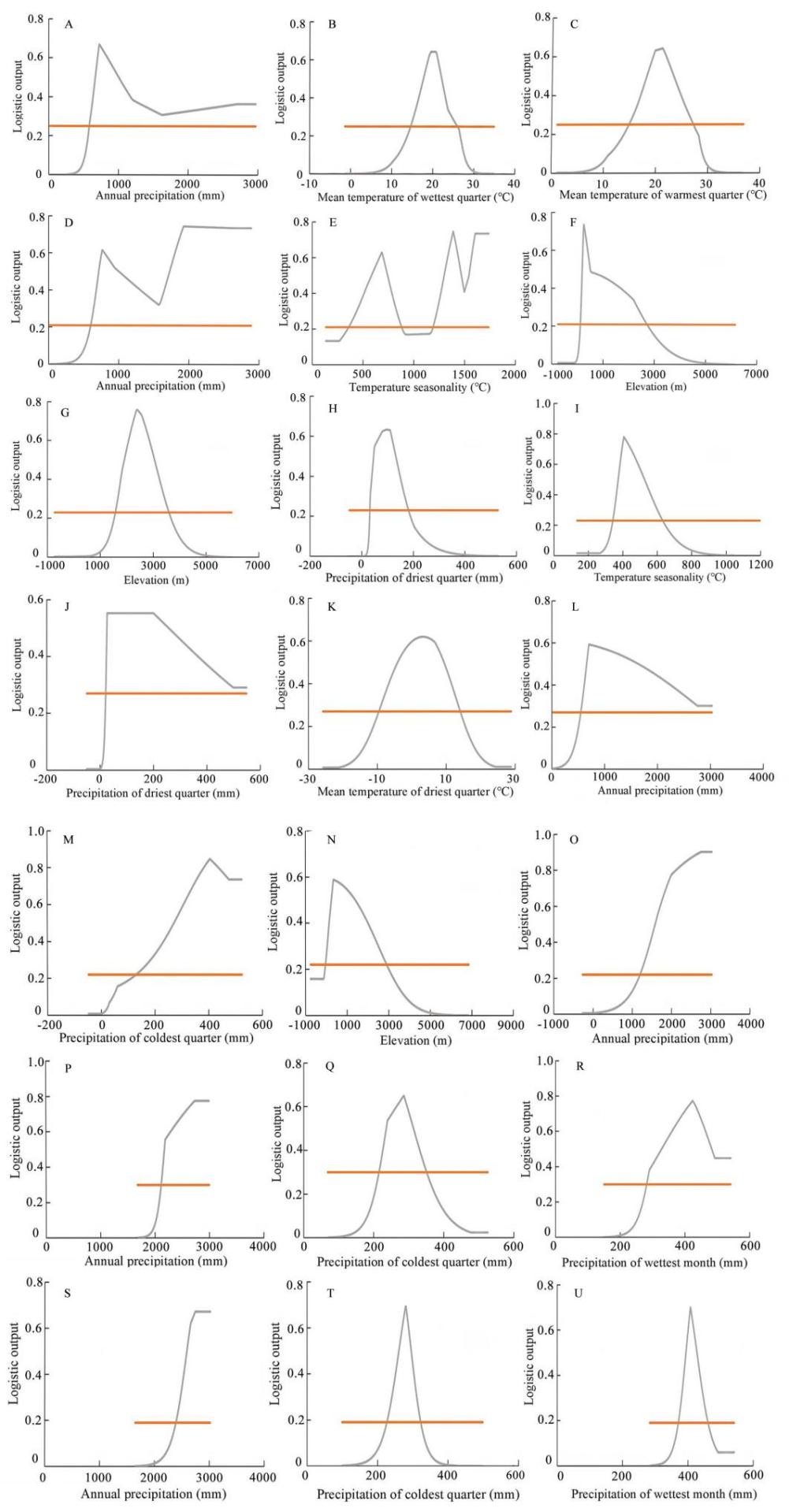


Supplementary Figure 2 Response curves of dominant environmental factors for *Astilbe*.


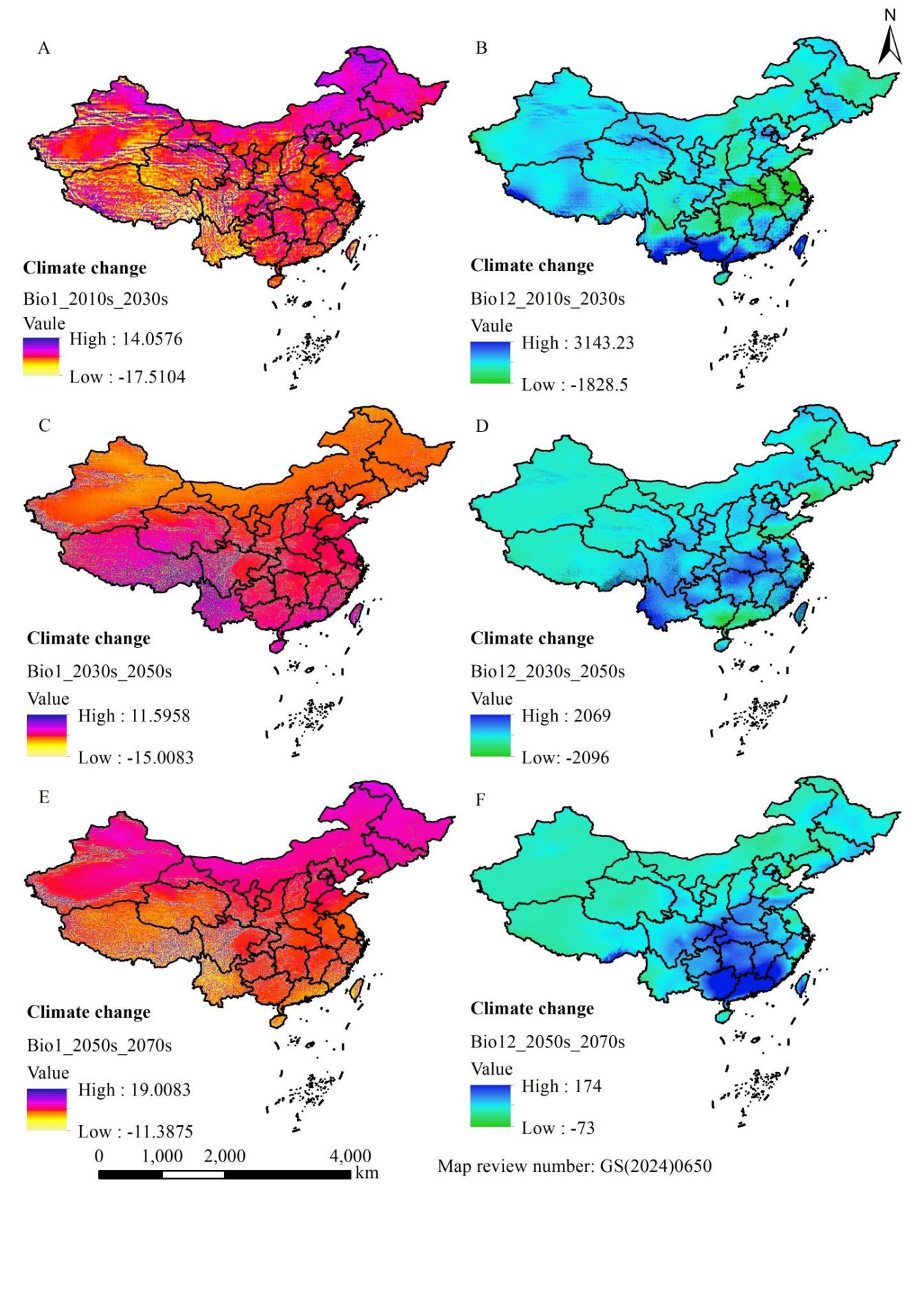


Supplementary Figure 3 Changes in average temperature and annual precipitation from the 2010s to the 2070s.

# Supplementary Tables

Supplementary Table 1 Field survey dataset of some *Astilbe* species.

| Species | Distribution pattern | Aspect | Slope gradient (°) | Slope position | Hsbitat | Elevation (m) | Survey date | Institution |
| --- | --- | --- | --- | --- | --- | --- | --- | --- |
| *A. chinensis* | Sparse | Northeast | 16.3 | Middle | Woodland | 1326.84 | 2022-07 | Hebei Forestry and Grassland Bureau |
|  | Sparse | Northwest | 8.3 | Lower | Woodland | 887.41 | 2022-07 | Hebei Forestry and Grassland Bureau |
|  | Clumped | North | 20.4 | Middle | Sparse woodland | 1167.94 | 2022-07 | Hebei Forestry and Grassland Bureau |
|  | Sparse | South | 6.3 | Lower | Woodland | 715.61 | 2022-07 | Hebei Forestry and Grassland Bureau |
|  | Clumped | North | 9 | Middle | Woodland | 1154.11 | 2022-07 | Hebei Forestry and Grassland Bureau |
|  | Scattered | Flat | 15 | Valley | Sparse woodland | 1185.3 | 2022-07 | Chifeng Forestry and Grassland Bureau |
|  | Clumped | Northwest | 6 | Lower | Woodland | 238.15 | 2022-06 | Heilongjiang Forestry and Grassland Bureau |
| *A. grandis* | Scattered | Flat | 2 | Lower | Woodland | 339.0 | 2022 - 08 | Heilongjiang Forestry and Grassland Bureau |
|  | Scattered | East | 5 | Upper | Woodland | 535.82 | 2023 - 06 | Songling Forestry Bureau |
|  | Clumped | North | 10 | Middle | Woodland | 548.73 | 2023 - 07 | Amuer Forestry Bureau |
|  | Patchy | Southwest | 4 | Middle | Woodland | 460.53 | 2023 - 07 | Amuer Forestry Bureau |
|  | Sparse | North | 12 | Middle | Woodland | 541.88 | 2021 - 06 | Heilongjiang Forestry and Grassland Bureau |
|  | Sparse | Flat | 3 | Flat | Grassland | 408.98 | 2022 - 08 | Heilongjiang Forestry and Grassland Bureau |
|  | Scattered | Flat | 3 | Flat | Woodland | 192.51 | 2022 - 08 | Heilongjiang Forestry and Grassland Bureau |
| *A. rivularis* | Scattered | South | 10 | Lower | Woodland | 2202.64 | 2023 - 05 | Gongshan County Forestry and Grassland Bureau |
|  | Sparse | Southeast | 10 | Lower | Woodland | 2284.13 | 2023 - 05 | Gongshan County Forestry and Grassland Bureau |
|  | Scattered | West | 21 | Middle | Woodland | 1764.55 | 2023 - 06 | Fugong County Forestry and Grassland Bureau |
|  | Scattered | Southwest | 25 | Middle | Woodland | 1994.46 | 2023 - 06 | Fugong County Forestry and Grassland Bureau |
|  | Scattered | Southwest | 20 | Middle | Woodland | 2406.5 | 2023 - 08 | Xiangyun County Forestry and Grassland Bureau |
|  | Sparse | Southwest | 30 | Upper | Woodland | 2799.2 | 2023 - 09 | Midu County Forestry and Grassland Bureau |
|  | Scattered | Northwest | 5 | Lower | Woodland | 2195.36 | 2024 - 03 | Qilin District Forestry and Grassland Bureau |
| *A. rubra* | Patchy | Southeast | 28 | Middle | Woodland | 1060.42 | 2023 - 08 | Hebei Forestry and Grassland Bureau |
|  | Patchy | East | 12 | Lower | Roadside | 473.0 | 2023 - 07 | Hebei Forestry and Grassland Bureau |
|  | Patchy | Southeast | 18 | Lower | Grassland | 757.13 | 2023 - 07 | Hebei Forestry and Grassland Bureau |
|  | Sparse | Flat | 5 | Valley | Alpine shrubland | 899.63 | 2023 - 07 | Hebei Forestry and Grassland Bureau |
|  | Sparse | South | 8 | Lower | Grassland | 639.23 | 2023 - 08 | Hebei Forestry and Grassland Bureau |
|  | Patchy | West | 12 | Lower | Grassland | 400.99 | 2023 - 08 | Hebei Forestry and Grassland Bureau |
|  | Patchy | West | 9 | Lower | Alpine shrubland | 496.4 | 2023 - 07 | Hebei Forestry and Grassland Bureau |
| *A. macrocarpa* | Scattered | East | 10 | Upper | Woodland | 2736.3 | 2023 - 08 | Yunnan Provincial Forestry and Grassland Bureau |
|  | Sparse | South | 24 | Lower | Woodland | 2312.5 | 2023 - 05 | - |
|  | Sparse | Northwest | 15 | Lower | Shrub forest | 1607.53 | 2024 - 05 | Xiangyun County Forestry and Grassland Bureau |

Supplementary Table 2 *Astilbe* sample collection dataset

| Species | Unscreened (no.) | | | Screened (no.) |
| --- | --- | --- | --- | --- |
|  | Field survey | CVH, GBIF | Literature record |  |
| 1. *chinensis* | 458 | 2999 | 13^[[1]](#footnote-0)^ | 615 |
| 1. *grandis* | 46 | 717 | 2^[[2]](#footnote-1)^ | 101 |
| 1. *rivularis* | 33 | 623 | - | 96 |
| 1. *rubra* | 9 | 1328 | - | 27 |
| 1. *macrocarpa* | 4 | 65 | - | 31 |
| 1. *longicarpa* | - | 1104 | - | 36 |
| 1. *macroflora* | - | 142 | - | 26 |
| Total | 540 | 6978 | 15 | 932 |

Supplementary Table 3 Environmental variables used in MaxEnt modeling for *Astilbe* species.

| Species | Environmental variables |
| --- | --- |
| 1. *chinensis* | bio2, bio4, bio8, bio10, bio12, bio13, bio15, bio18, clay, dem, sand |
| 1. *grandis* | bio4, bio5, bio10, bio12, bio13, bio17, clay, dem, sand |
| 1. *rivularis* | bio2, bio4, bio9, bio12, bio13, bio15, bio17, bio18, clay, dem, sand, silt |
| 1. *rubra* | bio9, bio12, bio15, bio17, bio18,clay, dem |
| 1. *macrocarpa* | bio3, bio6, bio10, bio12, bio13, bio14, bio15, bio16, bio18, bio19, dem |
| 1. *longicarpa* | bio4, bio7, bio12, bio13, bio19, dem |
| 1. *macroflora* | bio4, bio7, bio12, bio13, bio19, dem |

Supplementary Table 4 Model evaluation metrics and hyperparameter settings for *Astilbe* species.

| Species | AUC | TSS | T | RM | FC |
| --- | --- | --- | --- | --- | --- |
| 1. *chinensis* | 0.926 | 0.778 | 0.2432 | 1 | Hinge, product, linear, quadratic |
| 1. *grandis* | 0.969 | 0.844 | 0.1162 | 1 | Hinge, product, linear, quadratic |
| 1. *rivularis* | 0.987 | 0.941 | 0.1196 | 1 | Hinge, product, linear, quadratic |
| 1. *rubra* | 0.937 | 0.751 | 0.3337 | 1 | Hinge, linear, quadratic |
| 1. *macrocarpa* | 0.961 | 0.801 | 0.1284 | 1 | Hinge, linear, quadratic |
| 1. *longicarpa* | 0.998 | 0.999 | 0.3372 | 1 | Hinge, linear, quadratic |
| 1. *macroflora* | 0.999 | 0.999 | 0.1004 | 1 | Hinge, linear, quadratic |

Supplementary Table 5 Numerical ranges defining habitat suitability categories.

| Species | non-suitability  area | low-suitability  area | medium-suitability  area | high-suitability  area |
| --- | --- | --- | --- | --- |
| 1. *chinensis* | < 0.2432 | 0.2432 - 0.4955 | 0.4955 - 0.7477 | > 0.7477 |
| 1. *grandis* | < 0.1162 | 0.1162 - 0.4108 | 0.4108 - 0.7054 | > 0.7054 |
| 1. *rivularis* | < 0.1196 | 0.1196 - 0.4131 | 0.4131 - 0.7065 | > 0.7065 |
| 1. *rubra* | < 0.3337 | 0.3337 - 0.5558 | 0.5558 - 0.7779 | > 0.7779 |
| 1. *macrocarpa* | < 0.1284 | 0.1284 - 0.4189 | 0.4189 - 0.7095 | > 0.7095 |
| 1. *longicarpa* | < 0.3372 | 0.3372 - 0.5581 | 0.5581 - 0.7791 | > 0.7791 |
| 1. *macroflora* | < 0.1004 | 0.1004 - 0.4003 | 0.4003 - 0.7001 | > 0.7001 |

# Captions

In Supplementary Figures 1, the panel labels correspond to the following species: (A) *A. chinensis*, (B) *A. grandis*, (C) *A. rivularis*, (D) *A. rubra*, (E) *A. macrocarpa*, (F) *A. longicarpa*, and (G) *A. macroflora*.

In Supplementary Figure 2, (A - C): *A. chinensis*; (D - F): *A. grandis*; (G - I): *A. rivularis*; (J - L): *A. rubra*; (M - O): A. macrocarpa; (P - R): *A. longicarpa*; (S - U): *A. macroflora*. The red dashed lines indicate the medium-suitability habitat thresholds of each environmental factor for each species.

In Supplementary Figure 3, Changes in annual mean temperature (bio1) and annual precipitation (bio12) across different time periods. Panels A, C, and E show changes in bio1; Panels B, D, and F show changes in bio12. Change was calculated as the later period minus the earlier period: A and B, 2030s − 2010s; C and D, 2050s − 2030s; E and F, 2070s − 2050s. Temperature differences are in °C, and precipitation differences are in mm.

1. Yuan LJ, Yin H, Si Q, et al. Study on the HPLC fingerprint and pattern recognition of *Astilbe chinensis* from Hubei province[J]. West China Journal of Pharmaceutical Sciences, 2019, 34(4): 405-409.; Yin C, Tu X, Zhang JW, et al. Determination of bergenin in different parts of *Astilbe* *chinensis*[J]. China Pharmacist, 2018, 21(7): 1292-1295.; Wang J, Ma Y, Wang ZH, et al. Refluxing extraction of bergenin from *Astilbe* *chinensis*[J]. Journal of Northwest Forestry University, 2012, 27(4): 186-189.; Lei Bing. Studies on the Chemical Constituents and Pharmacological Activities of *Astilbe chinensis*. Master's Thesis, Jilin Agricultural University, 2012. [↑](#footnote-ref-0)
2. Zhang L, Zou J, Ye JH, et al. GC - MS and antioxidant capacity of volatile oil from the roots, stems andleaves of *Astilbe grandis*[J]. Central South Pharmacy, 2022, 20(12): 2767‑2771.; He K, Wu TT, Fan LL, et al. Chemical constituents from *Astilbe grandis*[J]. Chinese Traditional Patent Medicine, 2021, 43(1): 105-110. [↑](#footnote-ref-1)
